# Nuclear FOXP3 inhibits tumor growth and induced apoptosis in hepatocellular carcinoma by targeting c-Myc

**DOI:** 10.1038/s41389-020-00283-x

**Published:** 2020-10-28

**Authors:** Zhongqin Gong, Hao Jia, Jianqing Yu, Yi Liu, Jianwei Ren, Shengli Yang, Baoguang Hu, Liping Liu, Paul B. S. Lai, George Gong Chen

**Affiliations:** 1 Department of Surgery, Faculty of Medicine, Prince of Wales Hospital, The Chinese University of Hong Kong, Hong Kong, China; 2 Department of Thyroid and Breast Surgery, Peking University Shenzhen Hospital, Shenzhen, 518036 Guangdong China; 3 Guangdong Key Laboratory for Research and Development of Natural Drugs, Guangdong Medical University, Zhanjiang, 524025 Guangdong China; 4 Cancer Center, Union Hospital, Tongji Medical College, Huazhong University of Science and Technology, Wuhan, 430022 China; 5 Department of Gastrointestinal Surgery, the Affiliated Hospital of Binzhou Medical University, Binzhou, Shandong China; 6 Department of Hepatobiliary and Pancreas Surgery, the Second Clinical Medical College of Jinan University (Shenzhen People’s Hospital), Shenzhen, 524000 Guangdong Province China; 7 Department of Otorhinolaryngology, Head and Neck Surgery, Faculty of Medicine, Prince of Wales Hospital, The Chinese University of Hong Kong, Hong Kong, China

**Keywords:** Liver cancer, Cell signalling

## Abstract

The status of FOXP3 and its isoforms in hepatocellular carcinoma (HCC) is unclear. We aimed to investigate the expression and function of FOXP3 and its isoforms in HCC. The study was performed on 84 HCC patients, HCC cell lines and a mouse tumor model. The levels of FOXP3 and its isoforms were determined by nested PCR, quantitative real-time PCR and immunohistochemistry (IHC) staining. The correlation between their levels and clinicopathologic characteristics was analyzed. The full length of FOXP3 (FOXP3) and exon 3-deleted FOXP3 (FOXP3Δ3) were found to be the major isoforms in HCC. The levels of FOXP3Δ3 mRNA and protein in HCC tumor samples were not significantly different from their adjacent normal tissues. The high expression of FOXP3 protein in HCC patients showed a good overall survival. The overexpression of FOXP3 significantly reduced tumor cell proliferation, migration and invasion. The immunofluorescence result indicated that FOXP3 needed to be translocated into the nucleus to exert its inhibitory function. The luciferase assay demonstrated that FOXP3 could be synergistic with Smad2/3/4 to inhibit the oncogene c-Myc. The co-immunoprecipitation results further revealed that FOXP3 could interact with Smad2/3/4. The chromatin immunoprecipitation (ChIP) assay showed that both FOXP3 and Smad2/3/4 bound the promoter of the c-Myc to inhibit it. The in vivo mouse tumor model study confirmed the inhibitory effect of FOXP3. Collectively, the expression of tumor FOXP3 can inhibit the growth of HCC via suppressing c-Myc directly or indirectly via interacting with Smad2/3/4. Therefore, FOXP3 is a tumor suppressor in HCC.

## Background

The human FOXP3 gene has 12 exons, of which 11 exons are coded (E2-E12), but exon 1 is not coded. FOXP3 was first reported to be a critical regulator in the development of regulatory T cells^1,2^. Until recently, the expression of FOXP3 has been found in different cancer cells, including pancreatic carcinoma^3^, breast cancer^4,5^, prostate cancer^6^, thyroid cancer^7^. However, the results of these studies indicated that the role of FOXP3 in cancers was controversial.

Wang *et al*. found that FOXP3 was positive in 48% HCC tumor tissues by immunohistochemical (IHC) staining, but not in para-tumor tissues and normal liver tissues. The distribution of FOXP3 in HCC was similar to that of cirrhosis, but not to hepatitis caused by the hepatitis B virus (HBV), which suggested that FOXP3 was associated with a high risk of HCC^8^. PreS2, one of the crucial regulatory proteins encoded by HBV, could transactivate FOXP3 transcription and expression in malignant hepatocytes^9^. However, Shi *et al*. reported that the high expression of FOXP3 was significantly correlated with low serum α-fetoprotein (AFP) level, absence of vascular invasion, early TNM stage, better survival, and reduced recurrence^10^. Moreover, FOXP3 may suppress tumor progression via the TGF-β/smad2/3 signaling pathway in HCC^10^. Recently, Duan et al. claimed that FOXP3 up-regulated miR-198 expression by binding to its promoter, leading to the inhibition of proto-oncogene Myc in HepG2 cells^11^.

Currently, there is no explanation for the contradictory role of FOXP3 in HCC. However, the alternative RNA splicing and subcellular location of FOXP3 may offer an answer to the controversial effect. To date, five isoforms of FOXP3 have been identified, and their roles in cancers are diverse^12^. In this study, we aimed to study FOXP3 and its isoforms and explore their roles in HCC.

## Results

### The high expression of FOXP3 protein in HCC tissues indicated a good overall survival

The FOXP3 isoforms amplified by the nested PCR were confirmed by sequencing. We found that the major isoforms of FOXP3 in HCC tissue and adjacent normal tissue were the FOXP3 and FOXP3Δ3 (Supplementary Fig. S1). The quantitative real-time PCR results showed that the expression of FOXP3 was significantly higher in HCC tissues than the adjacent normal tissues but there was no significant difference in the expression of FOXP3Δ3 (Supplementary Fig. S2).

**Fig. 1:**
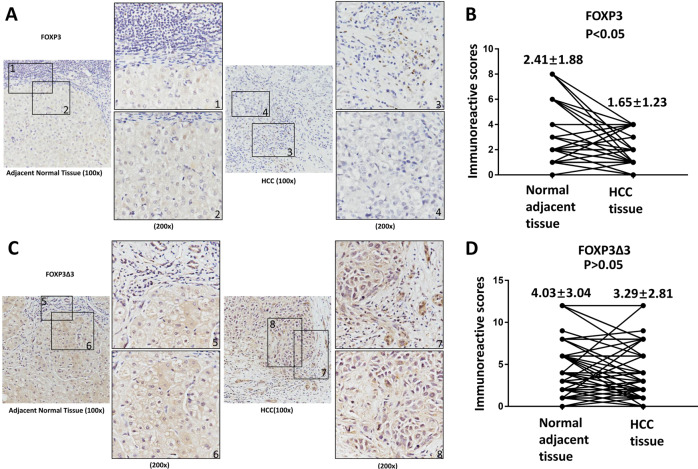
The expression of FOXP3 and FOXP3Δ3 in HCC tissue samples. **a** The representative image of the FOXP3 protein staining in the samples by FOXP3 150D/E4 antibody. The lymphocytes and the liver cells showed the FOXP3 positive staining. **c** The representative image of the FOXP3Δ3 protein staining in the samples by FOXP3Δ3 16J4G6 antibody. The lymphocytes and liver cells showed the FOXP3Δ3 positive staining. The levels of FOXP3 (**b**) and FOXP3Δ3 (**d**) in 84 paired HCC tissues and adjacent normal tissues were scored by the immune reactive score method and analyzed by paired *t*-test. Data were expressed as Mean ± SD. The correlation of FOXP3 and FOXP3Δ3 with clinicopathologic characteristics, please refers to supplementary Tables.

To determine the protein levels of FOXP3 and FOXP3Δ3 in the tissue samples, the IHC was performed. The IHC staining showed that the expression of FOXP3 and FOXP3Δ3 was positive in both hepatocytes and infiltrated lymphocytes (Fig. 1a, c), and the immune reactive score results indicated that the expression of FOXP3 was significantly higher in the hepatocytes of the adjacent normal tissues than the tumor tissues (Fig. 1b), whereas for FOXP3Δ3, there was no significant difference between the adjacent normal tissues and tumor tissues (Fig. 1d). Thus, the result of FOXP3 protein expression detected by IHC staining was differed from that of the FOXP3 mRNA level detected by the quantitative real-time PCR. Since the expression of FOXP3 in the infiltrated lymphocytes could not be excluded in the latter but was removed in the former, the FOXP3 expression measured by IHC staining should be more accurate or more reliable than quantitative real-time PCR.

To determine whether the levels of FOXP3 and FOXP3Δ3 were associated with patient prognosis, we analyzed the correlation between clinicopathologic characteristics and the FOXP3 or FOXP3Δ3 expression. The results showed that the high expression of FOXP3 was negatively correlated with tumor number (*P* = 0.015) and TNM stage (*P* = 0.040), suggesting the inhibitory role of FOXP3 in HCC (Supplementary Table S1). However, all clinicopathologic characteristics were not significantly correlated with FOXP3Δ3 expression (Supplementary Table S1).

To further explore whether FOXP3 had any prognostic value, we conducted Cox proportional hazard regression analysis of patients’ overall survival and disease-free survival. Both univariate analyses and multivariate analyses indicated that FOXP3 was an independent predictor for overall survival and disease-free survival of HCC patients (Supplementary Tables S2 and S3). Kaplan–Meier analysis also showed that patients with a high level of FOXP3 expression in tumors had longer overall survival and disease-free survival time (Supplementary Fig. S3,A, B).

Additionally, the univariate analyses and multivariate analyses showed that FOXP3Δ3 was not an independent predictor for overall survival and disease-free survival of HCC patients (Supplementary Tables S4 and S5). Therefore, in the following experiments, we focused on FOXP3 only.

### Ectopic expression of FOXP3 inhibited HCC cells proliferation, migration, and invasion

**Fig. 2:**
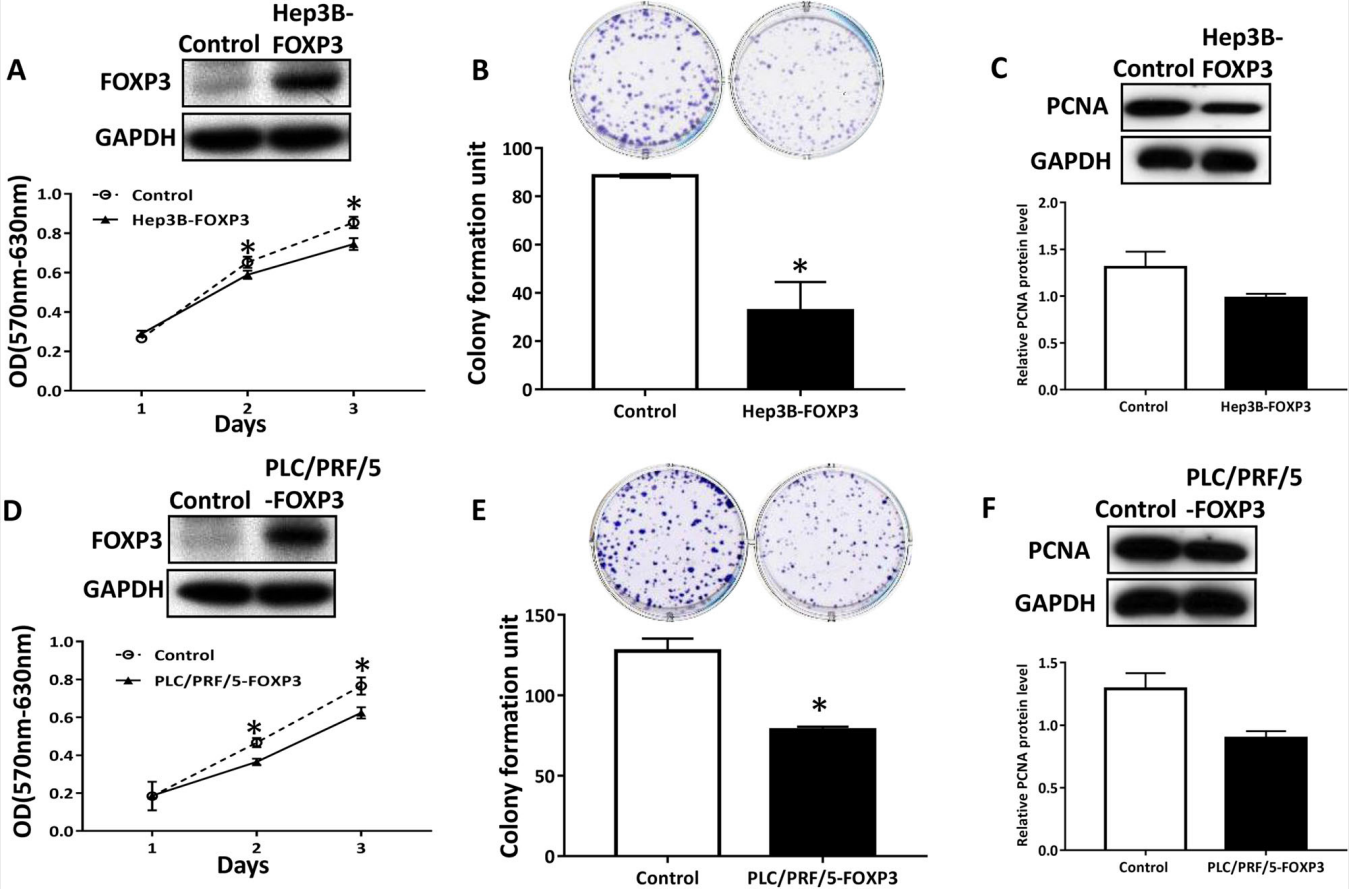
FOXP3 inhibited tumor growth in HCC. The effect of FOXP3 on cell viability was evaluated by MTT assay in Hep3B (**a**) and PLC/PRF/5 (**d**) cells. FOXP3 inhibited the colony formation in Hep3B (**b**) and PLC/PRF/5 (**e**) cells. **c**, **f** FOXP3 inhibited the expression of PCNA in Hep3B (**b**) and PLC/PRF/5 cells. *P < 0.05. These experiments were repeated 3 times.

To examine the effect of FOXP3 on the proliferation and migration of HCC cells, we first selected two cell lines with low FOXP3 expression, Hep3B and PLC/PRF/5 (Supplementary Fig. S3C), and generated FOXP3-overexpressing stable Hep3B and PLC/PRF/5 cell. The pcDNA3.1-FOXP3 plasmid was transfected by lipofectamine 2000, and pcDNA-3.1 was transfected as the control. 500 ng/ml of G418 was used to select a stable cell. We demonstrated that the overexpression of FOXP3 reduced cell viability in Hep3B and PLC/PRF/5 cells by MTT assay (Fig. 2a, d). FOXP3 inhibited colony formation in both Hep3B cells (Fig. 2b) and PLC/PRF/5 cells (Fig. 2e). To further confirmed the inhibitory effect of FOXP3, the proliferation marker PCNA level was determined by the western blot. The results indicated that FOXP3 decreased the PCNA protein level in both Hep3B cells (Fig. 2c) and PLC/PRF/5 cells (Fig. 2f).

We next examined the effect of FOXP3 on the migration and invasion ability of the Hep3B and PLC/PRF/5 by the trans-well assay and wound-healing assay. The results showed that the overexpression of FOXP3 inhibited the abilities of migration and invasion in both Hep3B cells (Supplementary Fig. S4) and PLC/PRF/5 cells (Supplementary Fig. S5).

### Ectopic expression of FOXP3 induced the apoptosis in HCC

**Fig. 3:**
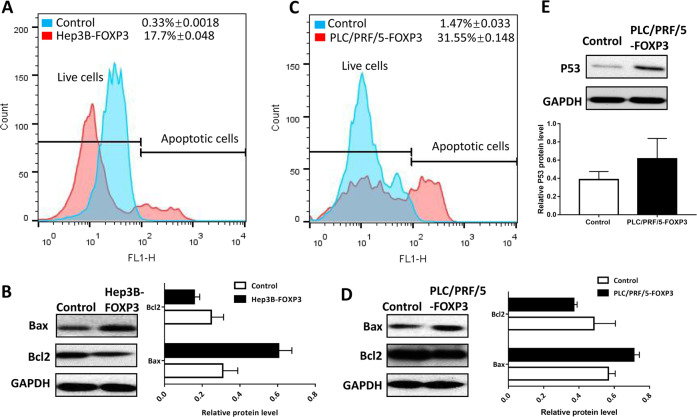
FOXP3 induced apoptosis in HCC cells. The proportion of apoptotic cells induced by FOXP3 in Hep3B (**a**) and PLC/PRF/5 (**c**) were evaluated by flow cytometry. **b**, **d**, **e** Effect of FOXP3 on pro-apoptotic maker Bax, anti-apoptotic marker Bcl2 and tumor suppressor p53. These experiments were performed in triplicate.

To determine the effect of FOXP3 on apoptosis, apoptosis assay by flow cytometry was performed. The results showed that there were significantly more apoptotic cells in Hep3B-FOXP3 (17.7% ± 0.048) than control cells (0.33 ± 0.001) (Fig. 3a). Besides, the anti-apoptotic marker Bcl-2 and pro-apoptotic marker Bax were determined by the western blot. The results showed that FOXP3 increased the expression of Bax, but decreased Bcl2 expression (Fig. 3b). These results supported that FOXP3 expression could cause the molecular changes in favor of the apoptosis induction in the HCC cells.

We observed similar results in the PLC/PRF/5 cells. The apoptotic population in PLC/PRF/5-FOXP3 (31.55%±0.148) was much higher than that in control cells (1.47 ± 0.033) (*P* < 0.05, Fig. 3c). The western blot results also showed that FOXP3 increased the expression of pro-apoptotic Bax, but decreased the anti-apoptotic Bcl2 expression in PLC/PRF/5 cells (Fig. 3d). In addition, the expression of p53 was increased by FOXP3 in PLC/PRF/ cells. These results suggested that FOXP3 was able to induce the apoptosis of the HCC cells.

### Down-regulation of FOXP3 in Huh7 promoted the growth of HCC cells

**Fig. 4:**
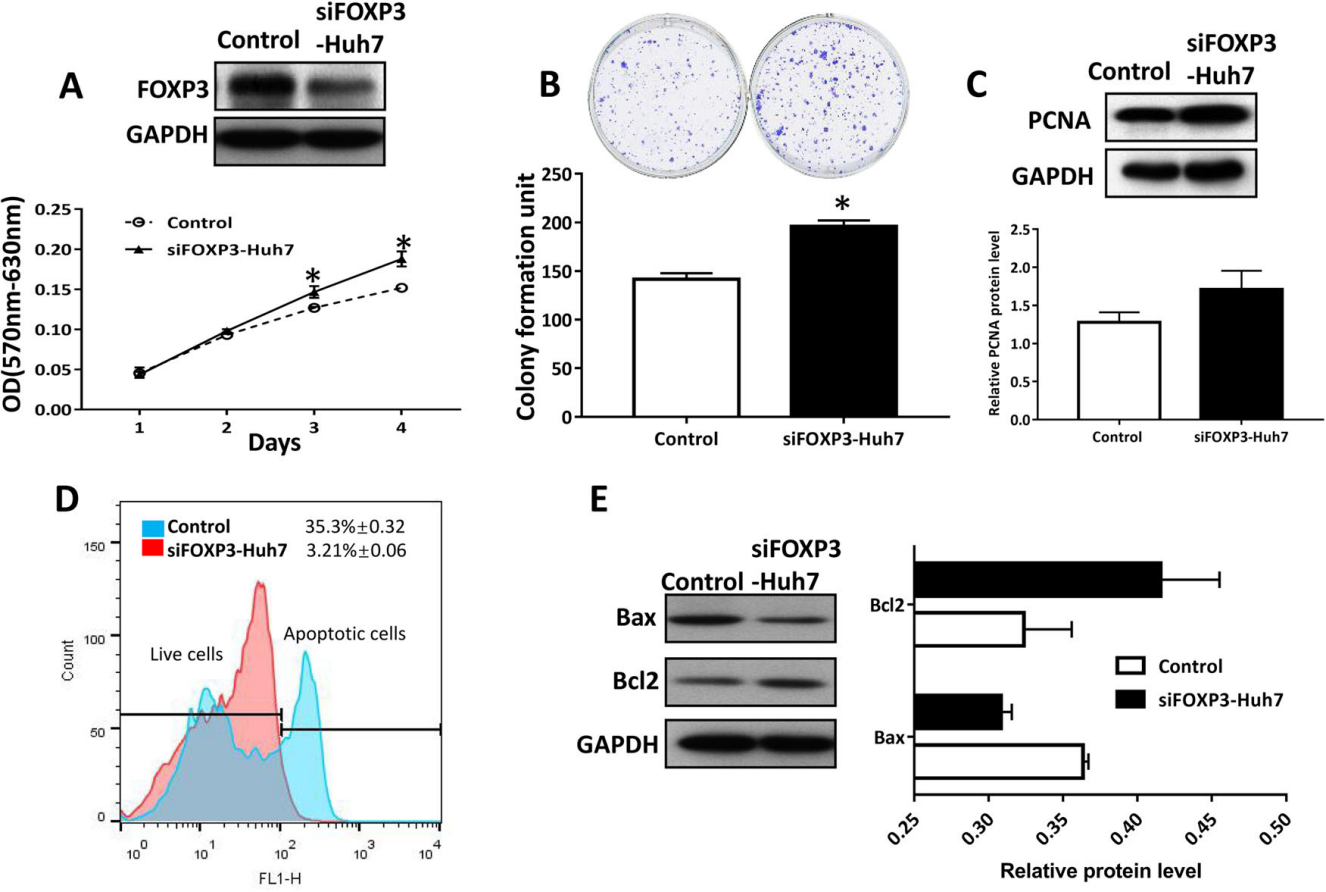
Knockdown of FOXP3 promoted tumor growth in Huh7 cells. Effect of FOXP3 knockdown on cell viability evaluated by MTT assay (**a**), colony formation (**b**), proliferation marker (**c**), apoptosis by flow cytometry (**d**) and apoptotic maker (**e**). *P < 0.05. These experiments were repeated 3 times.

To further confirm the effect of FOXP3 on HCC cells, we knocked down the FOXP3 expression by siRNA in Huh7 cell lines (Supplementary Fig. S3C). The results showed that the knockdown of FOXP3 significantly increased the cell viability in Huh7 cells (Fig. 4a), the abilities of colony formation (Fig. 4b), and the PCNA protein expression (Fig. 4c). The knockdown of FOXP3 was also shown to significantly promote HCC cell migration and invasion (Supplementary Fig. S6A, B). The flow cytometry result showed that after the downregulation of FOXP3 in Huh7 cells, the apoptotic population was obviously less than the control group (Fig. 4d). The Bcl2 protein level was increased, but the Bax protein level was decreased in siFOXP3-Huh7 cells (Fig. 4e). These results further confirmed that FOXP3 could inhibit the proliferation, migration, and invasion, but induce apoptosis in HCC cells.

### FOXP3 translocation into the nucleus was required to exert its inhibitory effect

P60 is a peptide that can inhibit the translocation of FOXP3 into the nucleus^13^. Using the cell immunofluorescence test, we confirmed that P60 could inhibit the translocation of FOXP3 into the nucleus in Hep3B-FOXP3 cells (Supplementary Fig. S6C) and that P60 reversed FOXP3-mediated inhibition of cell viability (Supplementary Fig. S6D). These results suggested that as a transcription factor and a tumor suppressor, the nuclear location of FOXP3 was required. In other words, the prevention of FOXP3 translocation into the nucleus can abolish the inhibitory function of FOXP3 in HCC cells.

### FOXP3 inhibited oncogene c-Myc

**Fig. 5:**
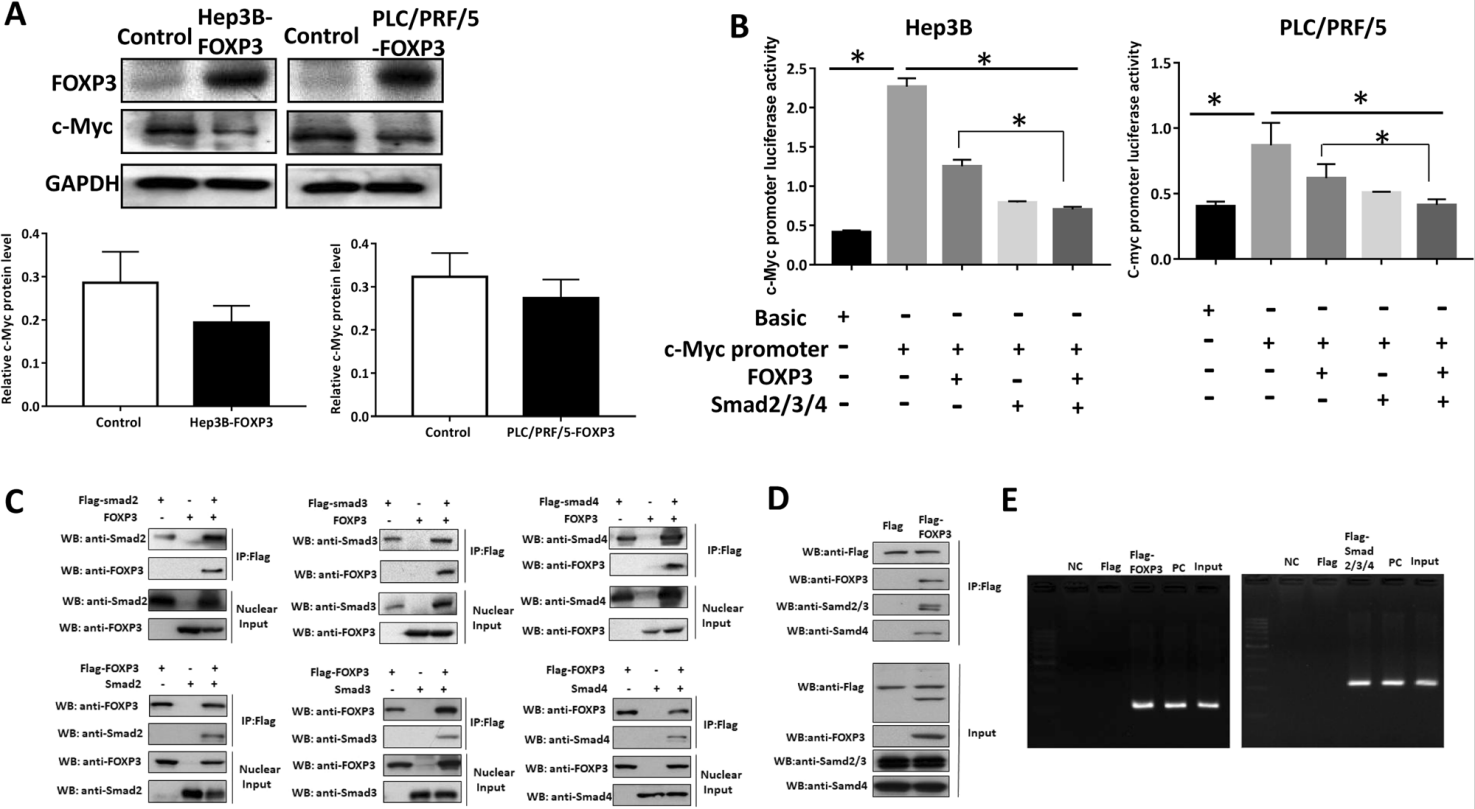
FOXP3 inhibited the oncogene c-Myc directly or indirectly via interacting with Smad2/3/4 in HCC cells. **a** Effect of FOXP3 on c-Myc expression in Hep3B and PLC/PRF/5 cells. **b** FOXP3 was directly or synergistic with Smad2/3/4 to inhibit the c-Myc promoter activity in Hep3B and PLC/PRF/5 cells. **c** FOXP3 interacted with Smad2, Smad3, and Smad4 in HEK-293T cells by co-immunoprecipitation. **d** FOXP3 interacted with Smad2, Smad3, and Smad4 in PLC/PRF/5 cells by co-immunoprecipitation. **e** FOXP3 and Smad2/3/4 could bind to the promoter of c-Myc by ChIP.

FOXP3 reduced the c-Myc expression in Hep3B and PLC/PRF/5 cells (Fig. 5a). To gain insights into the mechanism of the inhibitory effect of FOXP3 in HCC cells, RNA-sequencing was performed. We analyze the differential gene by the Reactome pathway. The Reactome pathway results indicated that FOXP3 might act on the TGFβ/Smads pathway to exert its effect (Supplementary Fig. S7), which is consistent with the early report^10^. Furthermore, oncogene c-Myc is one of the downstream genes in the TGFβ/Smads pathway^14^. Thus, FOXP3 may interact with Smads to target the c-Myc. To determine the effect of FOXP3 and Smad2/3/4 on the c-Myc promoter, the luciferase assay was performed. The plasmids were transfected into Hep3B and PLC/PRF/5 cells as indicated in figures. The results showed that both FOXP3 and smad2/3/4 could inhibit the promoter activity of the c-Myc. Furthermore, there is a synergistic effect of the FOXP3 and Smad2/3/4 when compared with FOXP3 only (Fig. 5b).

To further investigate the relationship of the FOXP3 and Smad2/3/4. The co-immunoprecipitation was performed in HEK-293T cells and PLC/PRF/5 cells. The results indicated that FOXP3 could form a complex with the Smad2, Smad3, and Smad4 (Fig. 5c, d), suggesting that there was crosstalk between the FOXP3 and Smad2/3/4.

We also investigated the correlation between FOXP3/Smad2/3/4 and the promoter of c-Myc by the ChIP assay. The results demonstrated that FOXP3 and Smad2/3/4 could bind the promoter of c-Myc in PLC/PRF/5 cells (Fig. 5e). These results suggested that FOXP3 could inhibit the oncogene c-Myc directly or indirectly via interacting with Smad2/3/4 in HCC cells.

### FOXP3 inhibited tumor growth in the mice

**Fig. 6:**
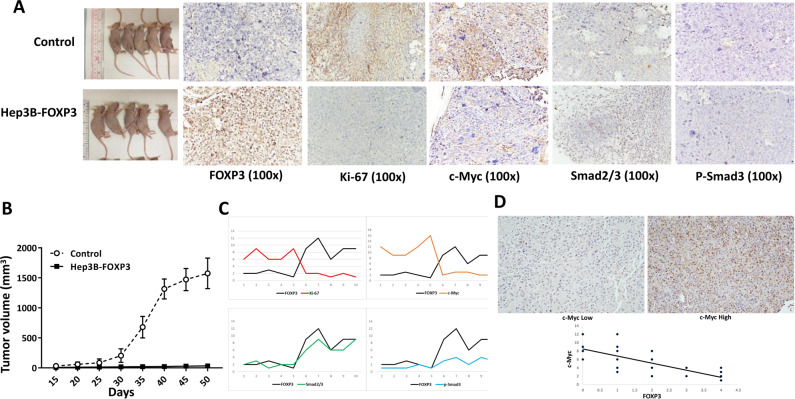
FOXP3 inhibited tumor growth in the tumor model of nude mice. **a** The representative image for tumor growth in nude mice and the detection of relevant proteins by IHC staining. **b** Subcutaneous tumor growth curve of Hep3B-FOXP3 in nude mice was compared with the control. The tumor volume in the FOXP3 group was much smaller than the control (*P* = 0.001). **c** The Pearson coefficient analysis of the correlation between FOXP3 and 4 other molecules: Ki-67 (-0.86), c-Myc (-0.87), Smad2/3 (0.89) and p-Smad3 (0.93), *P* < 0.05. **d** The expression of FOXP3 was negatively correlated with c-Myc in HCC tissues (*r* = −0.78, P < 0.05).

To confirm the in vitro results, xenograft assay was performed in nude mice. We found that the tumor volume formed by the Hep3B-FOXP3 was smaller than that form by Hep3B cells, indicating that FOXP3 inhibited tumor growth in vivo (*P* = 0.001, Fig. 6b). To examine the inhibitory effect of FOXP3 in vivo, the IHC was performed in the tumor tissues from the mice. The representative images were shown in Fig. 6a. The levels of FOXP3, Ki-67, c-Myc, Smad2/3, and p-Smad3 were quantitative by immune reactive scores. The Pearson correlation coefficient analyses were conducted to determine the correlation between FOXP3 and four other molecules (Ki-67, c-Myc, Smad2/3, and p-Smad3). The results showed that the expression of FOXP3 was negatively correlated with Ki-67 (*r* = −0.86, *P* < 0.05) and c-Myc (*r* = −0.87, *P* < 0.05), but positively correlated with Smad2/3 (*r* = 0.92, *P* < 0.05) and p-Smad3 (*r* = 0.93, *P* < 0.05).

We also investigated the correlation between FOXP3 and c-Myc in HCC tissues by IHC staining. The results showed that the expression of FOXP3 was negatively correlated with c-Myc (*r* = −0.78, *P* < 0.05) (Fig. 6d). These results support that FOXP3 acted as an HCC suppressor by inhibiting c-Myc expression.

## Discussion

As a FOX family transcription factor, FOXP3 mediates many gene expression. It is well known that FOXP3 serves as a critical regulator of CD4+ CD25+ regulatory T cell development and function^15^. Mutation of FOXP3 can result in immune dysregulation, polyendocrinopathy, enteropathy, X-linked (IPEX) syndrome, which is a rare, lethal disorder^16^. Besides, tumor-infiltrating lymphocytes represent the host immune response to cancer cells. A high prevalence of FOXP3+ regulatory T cells infiltrated HCC is thought to be an unfavorable prognostic indicator^17^. However, FOXP3Δ2Δ7 is unable to confer a suppressive ability to the regulatory T cells^18^. Therefore, the role of FOXP3+ regulatory T cells in the microenvironment of HCC is complicated.

In addition to the presence in lymphocytes, there are increasing publications showing the expression of FOXP3 in the cancer cells^12,19^, suggested that FOXP3 has a broader role than initial thoughts. However, the levels and roles of FOXP3 and its isoforms in HCC cells remains largely unknown or/and contradicted. Here, our data showed that hepatocytes could express FOXP3 and its isoforms but only full-length FOXP3 functioned as a tumor suppressor in HCC.

FOXP3 is consists of four functional domains: repressor domain, Zinc finger, leucine zipper, and FKH domain. Each domain has its specific roles, and alternative RNA splicing may lead to a fragmentary domain and affect its function (Supplementary Fig. S2A). To better know the exact role of FOXP3 in HCC, we focused on the FOXP3 and its isoforms in HCC. To date, five isoforms of the FOXP3 have been identified in different human cells and tissues^12^. In HCC, the full-length FOXP3 and exon3,4- deleted FOXP3 have been reported^10^. According to the TCGA database, four isoforms, FOXP3, FOXP3Δ3, FOXP3Δ8, and the unknown uc011mnb.2, were presented in HCC. In our study, we found that the FOXP3 (74%) and FOXP3Δ3 (76%) are the major isoforms in our HCC samples. Therefore, our experiment would focus on these two isoforms.

The detection methods selected, and the reagents used are critical for the study of tumor FOXP3 and isoforms in tissue samples. For the detection method, we found that IHC staining rather than PCR should be used. HCC tumor tissues usually contain the abundance of infiltrated lymphocytes that can express FOXP3. IHC staining can exclude these infiltrated lymphocytes, but PCR usually cannot. The selection of antibodies can significantly affect the result of the IHC staining. Different FOXP3 antibodies may generate different staining patterns^20^ leading to inconsistent results. For example, the antibody 236A/E7 can recognize both spliced and full-length forms of FOXP3 proteins and it may thus produce the results as a single form of FOXP3 in IHC. Moreover, since the function of the spliced FOXP3 isoforms is not the same as the full-length FOXP3, the significance of the results generated by non-differentiated antibodies is hard to be interpreted. In this study, we chose the 150D/E4 clone for the full-length FOXP3. This 150D/E4 maps to the exon 3 in human cells^21^, and thus it will not recognize FOXP3Δ3. For the detection of FOXP3Δ3, we selected the clone 16J4G6 that has been proved to specifically detect FOXP3Δ3^22^.

In this study, we demonstrated that the high expression of FOXP3 protein was correlated with less tumor number and early TNM stage, good overall survival and disease-free survival time, indicating that FOXP3 may act as a suppressor in HCC. However, there was no significant difference for the FOXP3Δ3 between the HCC tissues and adjacent normal tissues, and no clinicopathologic features were correlated with FOXP3Δ3 expression. The results may partially explain the previous controversial results^8–11^ as they did not exclude FOXP3Δ3 from the full-length FOXP3 or/and failed to separate the infiltrated lymphocytes from HCC tumor cells.

It is known that preS2 could transactivate FOXP3 transcription and expression in HepG2 cells^9^. In addition, the expression of FOXP3 is upregulated in HBV-positive cirrhosis^23^. However, our results did not show a significant correlation between the HBV infection (HBsAg) and the expression of FOXP3. This negative result may be due the small sample size as only 84 cases were studied. Nevertheless, the relationship between FOXP3 and hepatitis virus infection needs further investigation.

In this study, We selected two cell lines with low FOXP3 expression to perform the functional study, they are Hep3B and PLC/PRF/5. Both Hep3B and PLC/PRF/5 contain one or more integrated HBV DNA fragments^24^, but with low FOXP3 expression. Huh7 cells contains hepatitis C virus^25^ and with high FOXP3 expression. Therefore, we knocked down the expression of FOXP3 in Huh7 cells. Our functional study results showed that the ectopic expression of FOXP3 inhibited proliferation, migration and invasion of HCC cells (Hep3B and PLC/PRF/5), but increased the apoptotic population. It has been reported that FOXP3 is a key downstream regulator of p53-mediaetd cellular senescence in breast cancer cells^26^. Our data suggested that p53 may involve in the FOXP3-induced apoptosis in PLC/PRF/5 cells. These results were verified by the inhibitory experiments in Huh7 cells in which FOXP3 was downregulated by its siRNA. Therefore, the results of the functional tests supported the inhibitory role of FOXP3 in HCC.

Subcellular location can affect the function of FOXP3^27^. Similar to other transcription factors, FOXP3 needs to be translocated into the nucleus to execute its functions. P60, a peptide, is known to inhibit the translocation of FOXP3 into the nucleus^13^. Our immunofluorescence results confirmed that P60 was able to inhibit FOXP3 nuclear translocation in HCC cells, and the application of P60 could overcome FOXP3-mediated inhibition of tumor cell proliferation. These results have clearly indicated that the inhibition of FOXP3 nuclear translocation can abolish its inhibitory functions in HCC cells.

It is a common phenomenon that the expression of the proto-oncogene c-Myc is increased in HCC^28^, and that the level of c-Myc is correlated with a poor prognosis^29^. The inhibition of c-Myc appears to be a promising therapy against HCC^30^. We derived and reanalyzed the ChIP-sequencing results^10^, and identified that oncogene c-Myc was a gene directly targeted by FOXP3.

To explore the signaling pathway of tumor FOXP3, we found that FOXP3 could significantly downregulate oncogene c-Myc in HCC cells and our Reactome pathway analysis indicated that FOXP3 might act on the TGFβ/Smads pathway to exert its inhibitory function. Interestingly, c-Myc is one of the downstream molecules targeted by TGFβ/Smads^14^. Our luciferase, co-immunoprecipitation and ChIP assays indicated that either FOXP3 or Smad2/3/4 complex could interact with c-Myc protein or/and its promoter to inhibit c-Myc in HCC cells. And the administration of FOXP3 and Smad2/3/4 complex in combination synergistically inhibit c-Myc. The direct impact of either FOXP3 or Smad2/3/4 complex on c-Myc was supported by binding site JASPAR analysis^31^, showing that FOXP3 and Smad2/3/4 may bind to the TATA box of the c-Myc promoter. Further investigation is needed to specify the binding site. Nevertheless, our study has demonstrated that FOXP3 can inhibit c-Myc directly or indirectly via interacting with Smad2/3/4.

Finally, we verified the inhibitory effect of FOXP3 in vivo. A tumor formed by the FOXP3-overexpressing cells was much smaller than the control in the mouse tumor model, and the overexpression of FOXP3 induced the expression of smad2/3 and phosphorylated smad3 but inhibited the expression of c-Myc and Ki-67.

In conclusion, we have shown that there are two major forms of FOXP3, full-length FOXP3 and FOXP3Δ3 in HCC. However, it appears that FOXP3 rather FOXP3Δ3 functions as a tumor suppressor in HCC. FOXP3 arrests HCC growth by suppressing the proliferation, migration and invasion of the tumor cells and promotes apoptosis in HCC cells. Mechanically, we demonstrated that as a suppressor, FOXP3 could inhibit oncogene c-Myc directly or indirectly via interacting with Smad2/3/4.

## Methods

### Ethics statement

The human studies involved in this study were approved by the Joint Chinese University of Hong Kong- New Territories East Cluster Clinical Research Ethics Committee (CUHK-NTEC CREC). An informed consent for the human tissues for research purposes only was obtained from all patients recruited in this study. The animal work in this study was approved (17/088/MIS-5-B) by the animal experimentation ethics committee of CUHK.

### Clinical samples

A total of 84 pairs of HCC tissues and the corresponding adjacent para-tumor liver tissues were collected from patients who underwent surgery in the Prince of Wales Hospital. All the patients were diagnosed with HCC. These collected tissues were either fixed in 10% formalin for histological evaluation or snap-frozen in liquid nitrogen or stored at -80 °C until experimentation.

### Primers, plasmids, and antibodies

The primers used in this study were listed in Supplementary Table S6. The pcDNA3.1-FOXP3, pcDNA3.1, Flag-pcDNA3.1-FOXP3, Flag-pcDNA3.1 were produced in our Lab. CS2-flag-Smad2 (Addgen plasmid #14042) and CS2-flag-Smad3 (Addgen plasmid #14052) was a gift from Joan Massague, pcDNA3-Flag-Smad4 (Addgen plasmid #80888) was a gift from Aristidis Moustakas, Pbv-Luc (Addgen plasmid #16539) and c-Myc promoter del-1 (Addgen plasmid #16601) was a gift from Bert Vogelstein. SiRNA-FOXP3 (sc-43569) and siRNA-Control (sc-43569) were from Santa Cruz Biotechnology. The primary antibodies for western blot were as follows: FOXP3 (150D/E4, Invitrogen, 1:1000), FOXP3Δ3 (16J4G6, Novus,1:1000), PCNA (PC10, Santa Cruz, 1:2000), Bax (B-9, Santa Cruz, 1:1000), Bcl2 (C-2, Santa Cruz, 1:1000), p53 (DO-1, Santa Cruz, 1:1000), Smad2 (A-11, Santa Cruz, 1:1000), Smad3 (38-Q, Santa Cruz, 1:1000), Smad2/3 (A-3, Santa Cruz, 1:1000), p-Smad2(s467, Abcam, 1:1000), p-Smad3(1D9, Santa Cruz, 1:1000), Smad4 (B-8, Santa Cruz, 1:1000), c-Myc (9E10, Santa Cruz, 1:1000), and GAPDH (6C5, Santa Cruz, 1:2000). The primary antibodies for immunohistochemistry were listed below: FOXP3 (150D/E4, Invitrogen, 1:100), FOXP3Δ3 (16J4G6, Novus,1:100), Ki-67 (Ki67, Santa Cruz, 1:50), c-Myc (9E10, Santa Cruz, 1:100), Smad2/3 (A-3, Santa Cruz, 1:100), and p-Smad3 (Santa Cruz, 1:100).

### Nested PCR to determine the isoforms of FOXP3

RNA was isolated from 62 paired samples according to standard protocol (Trizol, Invitrogen), after reverse transcription, we performed two-round nested PCR (R004A, Takara). First-round PCR, we designed a pair of primers (FOXP3-1) to amplify the FOXP3 transcript. The primer (FOXP3-2) was used to amplify the region from exon 2 to exon 5, and primer (FOXP3-3) was used to amplify the region from exon 7 to exon 9. PCR products were cloned into T-A vectors and the sequence was checked by BGI HONGKONG company. Thus, the FOXP3 and its isoforms were confirmed by their sequences. All primers used in this study were listed in the Supplementary Table S6.

### Quantitative real-time PCR

After the determination and confirmation of the isoforms, we designed the specific primers to determine the levels of FOXP3 and FOXP3Δ3 in HCC. For the FOXP3, the forward primer was located in the junction between exon2 and exon3 (primer FOXP3-4). For the FOXP3Δ3, the forward primer was located in the junction between exon2 and exon4. The quantitative real-time PCR was performed to determine the mRNA level according to the protocol (RR820A, Takara) in the Quant-studio 12 K Flex Real-time PCR system.

### IHC staining

The IHC staining assay was performed according to the standard protocol on five-micrometer formalin-fixed paraffin sections by using the primary antibodies as indicated above. After staining, we invited a pathologist and an investigator who were blind to the study design to score the staining intensities according to the immunoreactive score system.

### Cell lines and culture conditions

Two HCC cell lines: Hep3B, PLCR/PRF/5, and HEK293T cell line were obtained from the American type culture collection. Huh7 was obtained from the Japanese Collection of Research Bioresource Cell Bank (JCRB0403). All cells were cultured in DMEM with 10% fetal bovine serum and 1% antibiotic at 37°C in humidified air with 5% CO_2_.

### The generation of FOXP3 overexpression stable cells

Hep3B and PLC/PRF/5 cell lines were used to generated FOXP3 overexpression stable cell lines. The plasmid pcDNA3.1-FOXP3 and corresponding vectors were transfected with lipofectamine 2000 transfection reagent (Invitrogen) according to the manufacture’s protocol. After transfected, the cells were selected by G418 at 500 ng/ml for two weeks.

### Knockdown FOXP3 by siRNA

The expression of FOXP3 was knocked down by siRNA in Huh7. siRNA-FOXP3 and siRNA-control were transfected on to the cells with the aid of lipofectamine 2000.

### Western blot

The total protein of the cells was isolated by the RIPA lysis buffer (20-188, Merck, Germany) with the protease inhibitor cocktail (Roche, Basel, Switzerland), and phenylmethylsulphonyl fluoride (Roche). The Bio-Rad Bradford protein assay was used to determine protein concentration. Before electrophoresis, the protein was denatured with the 1× protein loading dye at 95°C for 10 min. Samples containing 20 μg of protein were electrophoresed. Then, the protein was transferred on to 0.22 μm polyvinylidene difluoride (PVDF, BioRad). After transferred, the PVDF membrane was blocked in TBST containing 5% non-fat milk for 1 h at room temperature with shaking, and followed by incubating with primary antibodies at the indicated concentration at 4 °C overnight with shaking. We washed the membrane with the TBST and incubated with the corresponding secondary antibodies. The enhanced chemiluminescent substrate and western blot film plates from Kodak (Rochester, NY) were used to detect protein levels on the membranes in the darkroom. The protein expression level was semi-quantified by the internal control GAPDH (Santa Cruz Biotechnology). Then the grayscale ratio was determined by Adobe Photoshop CS5 (San Jose, CA). All experiments were performed in triplicate.

### Cell proliferation assays

An MTT kit (Sigma) was used to assay the cell proliferation according to the manufacturer’s instruction. FOXP3-overexpressing cells, Hep3B and PLC/PRF/5, and FOXP3-knockdown cells Huh7 and their corresponding control cells were seeded into 96-well plates, and cultured for different periods(24 h, 48 h, and 72 h). 10 µl 5 mg/ml MTT was added to each well, and the OD570 and OD630 were determined after 4 hours of incubation. The cell viability was represented by OD570 minus OD630.

### Colony formation assays

After 1000 FOXP3-overexpressing cells, Hep3B and PLC/PRF/5, and FOXP3- knockdown cells Huh7 and their corresponding control cells were seeded into 6-well plate for 14 days, colonies containing more than 50 cells were counted.

### Migration and invasion assays

At 24 h after transfection, approximately 5 × 10^3^ transiently transfected and the control cells were suspended in 200 μl of serum-free media and cultured in the upper chamber (8 μm pore size, Millipore) for the migration assay. For the invasion assay, the chamber was coated with 20% Matrigel (Sigma) 24 h before culturing with cells. 800 ml of the cell growth medium was filled within the lower chambers. After 48-hour incubation, the cells that had migrated or invaded through the membrane from the upper chamber to the lower chamber were fixed by the 4% paraformaldehyde and stained with 0.05% crystal violet (Sigma). The cells in the below of chamber surface were photographed, and the cells in three random fields were counted.

### Apoptosis assay

Apoptosis was detected using the apoptosis Assay Kit (ab-176749 Abcam) following the manufacturer’s protocol by flow cytometry.

### Immunofluorescence assay

After the treatment with peptide 60 for 24 h, cells were fixed at room temperature in 3% paraformaldehyde (PFA), permeabilized with 0.5% Triton X-100, and blocked with BSA. After blocked, the cells were incubated with primary antibodies (FOXP3, Invitrogen, 1:100) and fluorochrome-conjugated secondary antibodies (goat α mouse-Cy2/Cy3, Jackson ImmunoResearch, 1:500) for 1 h each. The nuclei of the cells were stained by DAPI. Stained cells were analyzed with a fluorescence microscope (Carl Zeiss).

### Dual-luciferase reporter assay

Hep3B and PLC/PRF/5 cells were transfected or co-transfected with various plasmids, as indicated in the figures. Cells were collected 48 h after transfection, and luciferase activities were detected by the dual-luciferase reporter assay kit (Promega, Fitchburg, WI). The luciferase activity was normalized to the control Renilla.

### Co-immunoprecipitation

After HEK293T or PLC/PRF/5 cells were transfected or co-transfected with plasmids, as shown in the figures for 24 h, the nuclear protein was extracted for immunoprecipitation. The ANTI-FLAG® M2 Affinity Gel was added into the lysis and incubated with continuing slow rotation at 4 °C overnight. After washing and denatured, the samples were used for western blot analysis.

### ChIP assay

ChIP assay was performed with a Magna ChIP kit (Merck, Darmstadt, Germany) according to the manufacturer’s instructions. PLC/PRF/5 cells were sonicated to shear the chromatin to a manageable size. The antibody for Flag antibodies (Invitrogen) was used for immunoprecipitation. End-point PCR was conducted to detect targeted DNA.

### Xenograft assay

Female nude mice (4–6 weeks old, weighing 16–20 g) were provided by Laboratory Animal Service Center of CUHK. The mice were free to access to food and water on a 12-h light, 12-h dark cycle. Ten female nude mice were randomly subjected to two groups (five mice per groups) that were subcutaneously injected with 1 × 10^6^ Hep3B-FOXP3 and Hep3B-control cells, respectively. The tumor size was measured every 3 days for up to 30 days by a micrometer. The tumor volume was calculated by length × width × width/2. After 30 days, the mice were euthanized by cervical dislocation, and the tumors were collected for further investigation. No adverse event of the animals was observed in this study. The experiments were repeated twice. All experimental procedures were approved by the Animal Ethics Committee of the Chinese University of Hong Kong.

### Statistical analysis

In this study, the continuous data were presented as the median ± SD, and discrete variables were presented as absolute values and relative frequencies. We used the paired t-test to compare the expression levels of FOXP3 and FOXP3Δ3 in tumor tissues and adjacent normal tissues. The clinicopathologic features were compared using Pearson’s chi-squared test or Fisher’s exact test. Kaplan–Meier plots were used for OS and DFS rates, then compared with the log-rank test. Univariable or multivariable Cox proportional hazard regression was performed to evaluate the predictive values of FOXP3 and other clinicopathologic features. The independent Student’s t-test was used to compare colony formation and gene expression between two groups. Repeated Measures ANOVA was used to compare the tumor growth rate between two groups in the in vivo assay. All the tests were performed by SPSS 22.0. P-values less than 0.05 were considered statistically significant.

## Supplementary information

Supplement tables

Supplement Figures

## Data Availability

All data generated or analyzed during this study are included in this published article and its supplementary information files.
