# Supplementary material for: Nuclear FOXP3 inhibits tumor growth and induced apoptosis in hepatocellular carcinoma by targeting c-Myc
Source: Oncogenesis. 2020 Oct 28;9(10):97. doi: 10.1038/s41389-020-00283-x (PMC7595121; doi:10.1038/s41389-020-00283-x)
Supplement: Supplementary file 1 — Supplement tables [file 41389_2020_283_MOESM1_ESM.pdf]

Table S1. Correlation between FOXP3 and FOXP3Δ3 expression and clinicopathologic characteristics

	FOXP3 Staining			FOXP3Δ3 staining		
	Low(n=66)	High(n=18)	P	Low (n=63)	High(n=21)	P
Gender						
Female	10	4	0.487	13	1	0.173
Male	56	14		50	20	
Age (years)						
≤50	27	7	0.877	25	9	0.797
>50	39	11		38	12	
Serum AFP (ng/ml)						
NA	31	6	0.531	23	14	0.055
≤20	11	3		12	2	
>20	24	9		28	5	
Tumor number						
Single	34	15	0.015*	41	10	0.156
Multiple	32	3		22	11	
Tumor Size (cm)						
≤5	43	15	0.139	47	11	0.056
>5	23	3		16	10	
Vascular invasion						
No	53	16	0.507	54	15	0.188
Yes	13	2		9	6	
Liver cirrhosis						
No	27	6	0.56	21	12	0.053
Yes	39	12		42	9	
HBsAg						
NA	8	1	0.552	6	3	0.605
Negative	7	1		7	1	
Positive	51	16		50	17	
TNM stage						
I	34	14	0.04*	40	10	0.199
II/III	32	4		23	11	
Tumor differentiation						
NA	13	2	0.701	12	3	0.46
Well/moderate	43	13		43	13	
Poor	10	3		8	5	

Table S2. Cox proportional Hazard regression analysis of patients' overall survival

	Univariable				Multivariable			
		95%CI				95%CI		
Varibles	HR	Lower	Upper	P	HR	Lower	Upper	P
Gender (male vs female)	1.629	0.8	3.318	0.178	1.286	0.603	2.743	0.514
Age (years) >50 vs ≤50	2.087	1.19	3.66	0.01*	1.889	1.013	3.525	0.046*
AFP(ng/ml) >20 vs ≤ 20	0.531	0.294	0.958	0.036*	0.842	0.61	1.162	0.294
Number of tumor multiple vs single	1.304	0.776	2.191	0.316	4.065	0.413	40.008	0.229
Tumor size(cm) >5 vs ≤ 5	1.151	0.666	1.989	0.613	1.095	0.57	2.102	0.786
Vascular invasion Yes vs No	1.759	0.903	3.427	0.097	2.582	1.069	6.238	0.035*
Liver cirrhosis Yes vs No	1.735	1.017	2.961	0.043*	1.87	1.018	3.437	0.044*
HBsAg Positive vs Negative	1.416	0.425	4.717	0.571	1.396	0.857	2.724	0.18
TMNstahge II/III vs I	1.149	0.684	1.929	0.599	0.22	0.021	2.35	0.21
Tumor differentiation Poor vs well/moderate	0.945	0.342	2.611	0.913	1.047	0.628	1.743	0.861
FOXP3 expression High vs Low	0.441	0.228	0.853	0.015*	0.43	0.211	0.875	0.02*

Table S3. Cox proportional Hazard regression analysis of patients' recurrence-free survival

	Univariable				Multivariable			
		95%CI				95%CI		
Varibles	HR	Lower	Upper	P	HR	Lower	Upper	P
Gender (male vs female)	1.712	0.84	3.487	0.139	1.348	0.64	2.841	0.432
Age (years) >50 vs ≤50	1.958	1.113	3.446	1.958	1.826	0.978	3.41	0.059
AFP(ng/ml) >20 vs ≤ 20	0.488	0.27	0.883	0.018*	0.752	0.545	1.038	0.83
Number of tumor multiple vs single	1.237	0.737	2.077	0.42	2.587	0.158	42.257	0.505
Tumor size(cm) >5 vs ≤ 5	1.196	0.692	2.067	0.521	0.921	0.488	1.739	0.8
Vascular invasion Yes vs No	2.131	1.065	4.262	0.032*	2.517	1.038	6.106	0.41
Liver cirrhosis Yes vs No	1.667	0.978	2.844	0.061	1.622	0.893	2.947	0.112
HBsAg Positive vs Negative	1.399	0.556	3.52	0.475	1.202	0.739	1.954	0.459
TMNstahge II/III vs I	1.18	0.703	1.982	0.531	0.321	0.018	5.607	0.436
Tumor differentiation Poor vs well/moderate	1.802	0.848	3.83	0.126	1.223	0.745	2.01	0.426
FOXP3 expression High vs Low	0.477	0.247	0.922	0.028*	0.459	0.228	0.925	0.029*

Table S4. Cox proportional Hazard regression analysis of patients' overall survival

Varibles	Univariable				Multivariable			
		95%CI				95%CI		
	HR	Lower	Upper	P	HR	Lower	Upper	P
Gender (male vs female)	1.629	0.8	3.318	0.178	1.404	0.657	2.999	0.381
Age (years) >50 vs ≤50	2.087	1.19	3.66	0.01*	1.886	1.019	3.492	0.043*
AFP(ng/ml) >20 vs ≤ 20	0.531	0.294	0.958	0.036*	0.825	0.593	1.146	0.25
Number of tumor multiple vs single	1.304	0.776	2.191	0.316	5.62	0.597	52.869	0.131
Tumor size(cm) >5 vs ≤ 5	1.151	0.666	1.989	0.613	1.239	0.635	2.418	0.529
Vascular invasion Yes vs No	1.759	0.903	3.427	0.097	2.75	1.083	6.985	0.033*
Liver cirrhosis Yes vs No	1.735	1.017	2.961	0.043*	1.842	0.981	3.457	0.057
HBsAg Positive vs Negative	1.416	0.425	4.717	0.571	1.348	0.832	2.186	0.225
TMNstahge II/III vs I	1.149	0.684	1.929	0.599	0.147	0.014	1.533	0.109
Tumor differentiation Poor vs well/moderate	0.945	0.342	2.611	0.913	1.075	0.64	1.808	0.784
FOXP3Δ3 expression High vs Low	1.314	0.731	2.361	0.362	1.312	0.678	2.54	0.42

Table S5. Cox proportional Hazard regression analysis of patients' recurrence-free survival

	Univariable				Multivariable			
		95%CI				95%CI		
Varibles	HR	Lower	Upper	P	HR	Lower	Upper	P
Gender (male vs female)	1.712	0.84	3.487	0.139	1.351	0.637	2.865	0.433
Age (years) >50 vs ≤50	1.958	1.113	3.446	1.958	1.817	0.985	3.352	0.056
AFP(ng/ml) >20 vs ≤ 20	0.488	0.27	0.883	0.018*	0.74	0.532	1.028	0.072
Number of tumor multiple vs single	1.237	0.737	2.077	0.42	3.228	0.189	55.237	0.419
Tumor size(cm) >5 vs ≤ 5	1.196	0.692	2.067	0.521	1.052	0.553	2.002	0.877
Vascular invasion Yes vs No	2.131	1.065	4.262	0.032*	2.768	1.086	7.054	0.033*
Liver cirrhosis Yes vs No	1.667	0.978	2.844	0.061	1.669	0.903	3.087	0.102
HBsAg Positive vs Negative	1.399	0.556	3.52	0.475	1.129	0.698	1.842	0.621
TMNstahge II/III vs I	1.18	0.703	1.982	0.531	0.238	0.013	4.422	0.336
Tumor differentiation Poor vs well/moderate	1.802	0.848	3.83	0.126	1.259	0.762	2.028	0.369
FOXP3Δ3 expression High vs Low	1.289	0.718	2.316	0.396	1.212	0.632	2.322	0.563

Table S6. Primers used in this study

Primers	Sequence(5'-3')	Application
FOXP3-1 For	AGTTTCCCACAAGCCAGGCT	Nested PCR Round1
FOXP3-1 Rev	TGGGGAGCTCGGCTGCAGTT	Nested PCR Round1
FOXP3-2 For	TGCCTCCTCTTCTTCCTTGA	Nested PCR Round2
FOXP3-2 Rev	ATTTGGGAAGGTGCAGAGC	Nested PCR Round2
FOXP3-3 For	CTGCCAGGCGGACCATC	Nested PCR Round2
FOXP3-3 Rev	GAATGTGCTGTTTCCATGGCT	Nested PCR Round2
FOXP3-4 For	GGCACTCCTCCAGGACAG	Real-time PCR
FOXP3-4 Rev	GCTGATCATGGCTGGGCTCT	Real-time PCR
FOXP3Δ3 For	CAGCTGCAGCTCTCAACGGTG	Real-time PCR
FOXP3Δ3 Rev	TGAGGAAGTCCTCTGGCTCT	Real-time PCR
ACTIN-For	AGGATGCAGAAGGAGATCAC	Real-time PCR
ACTIN-Rev	TGTAACGCAACTAAGTCATAG	Real-time PCR
Chip-For	AGTACTGCTACGGAGGAGCA	Chip assay
Chip-Rev	GCGGAGATTAGCGAGAGAGG	Chip assay
