# Supplementary material for: Nuclear FOXP3 inhibits tumor growth and induced apoptosis in hepatocellular carcinoma by targeting c-Myc
Source: Oncogenesis. 2020 Oct 28;9(10):97. doi: 10.1038/s41389-020-00283-x (PMC7595121; doi:10.1038/s41389-020-00283-x)
Supplement: Supplementary file 2 — Supplement Figures [file 41389_2020_283_MOESM2_ESM.pdf]

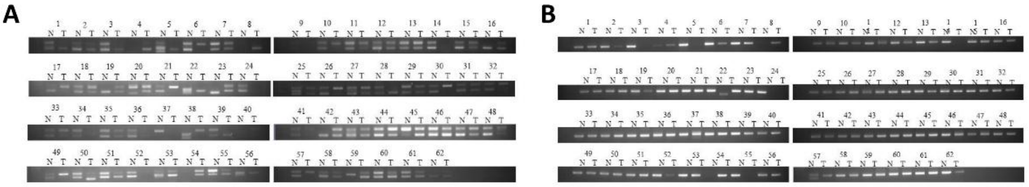

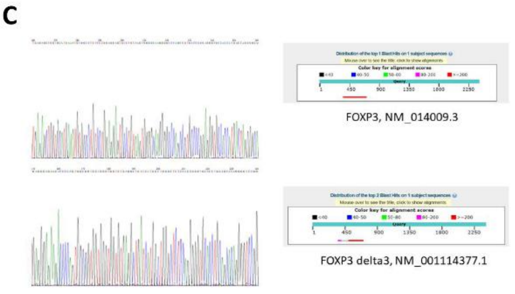

D

	FOXP3FL	FOXP3D3	FOXP3D3D4	FOXP3D8	FOXP3D3D8
N	48/62	54/62	4/62	1/62	1/62
T	46/62	47/62	1/62	0/62	1/62

Figure S1. FOXP3 and FOXP3Δ3 are the major isoforms in HCC tissues and adjacent normal tissues. A Nested PCR for detection of FOXP3, FOXP3Δ3 and FOXP3Δ4. B Nested PCR for detection of FOXP3 and FOXP3Δ8. C The sequencing results for the nested PCR products. D The statistics of FOXP3 isoforms in 62 paired HCC tissues and adjacent normal tissues.

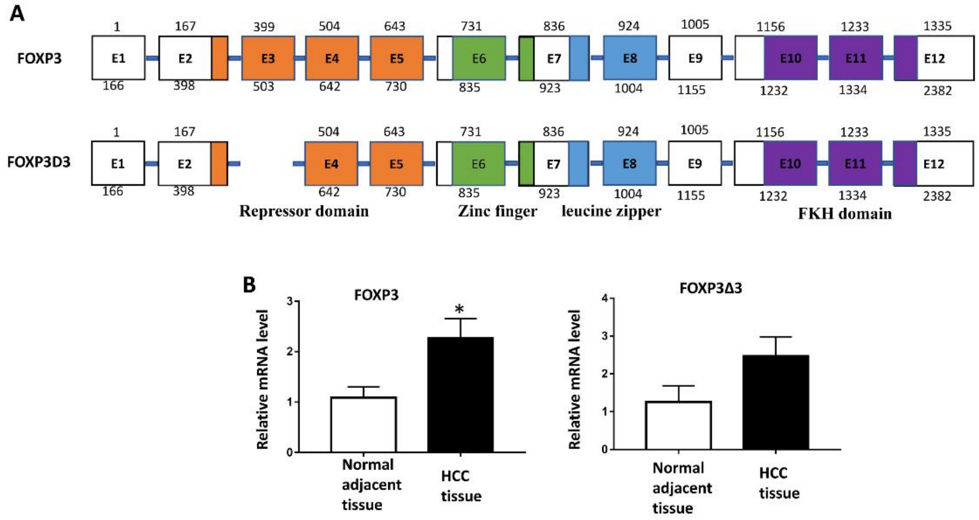

Figure S2. A Schematic representation of the structure of FOXP3 and FOXP3Δ3. B The mRNA level of FOXP3 and FOXP3Δ3 in HCC tissues and normal adjacent tissues, *P<0.05.

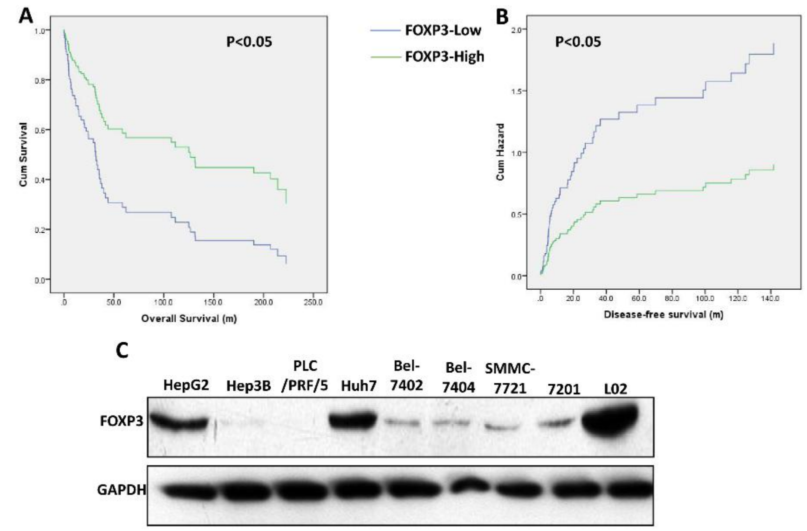

Figure S3. A The high level of FOXP3 expression in tumors had longer overall survival time(A) and disease-free survival time, showed as cum Hazard (B). C FOXP3 expressed in HCC cell lines by western blot.

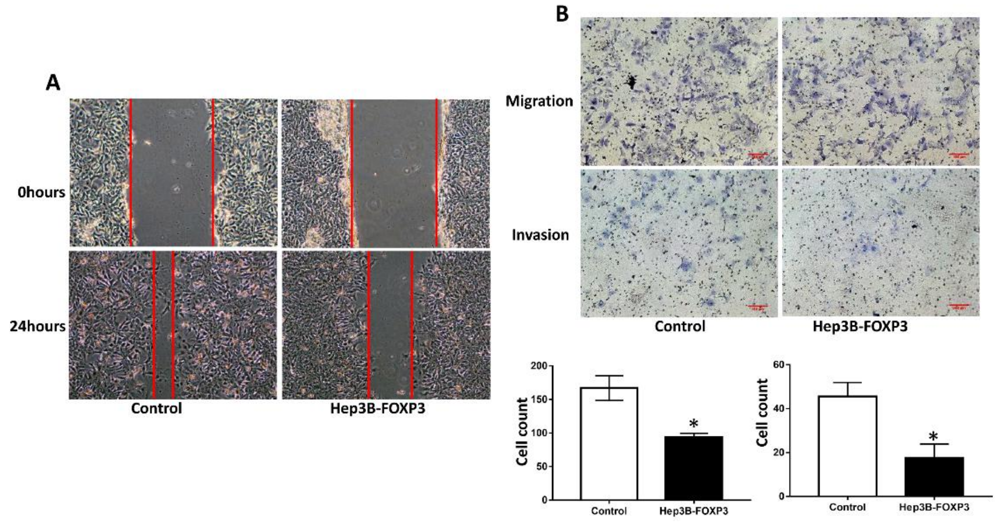

Figure S4. FOXP3 inhibits the ability of migration and invasion in Hep3B cells. A Effect of FOXP3 on the migration of Hep3B by wound healing assay. B Effect of FOXP3 on migration and invasion of Hep3B by trans-well assay. The upper panel was the representative image. The left lower panel showed the number of migrative cells. The right lower panel showed the number of invasive cells. *P<0.05.

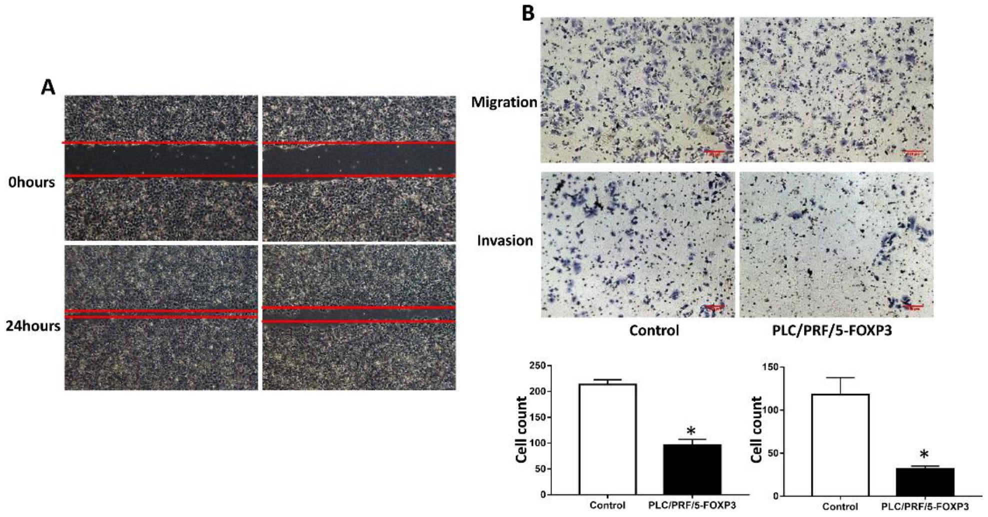

Figure S5. FOXP3 inhibits the ability of migration and invasion in PLC/PRF/5 cells. A Effect of FOXP3 on the migration of PLC/PRF/5 by wound healing assay. B Effect of FOXP3 on migration and invasion of PLC/PRF/5 by trans-well assay. The upper panel was the representative image. The left lower panel showed the number of migrative cells. The right lower panel showed the number of invasive cells. *P<0.05.

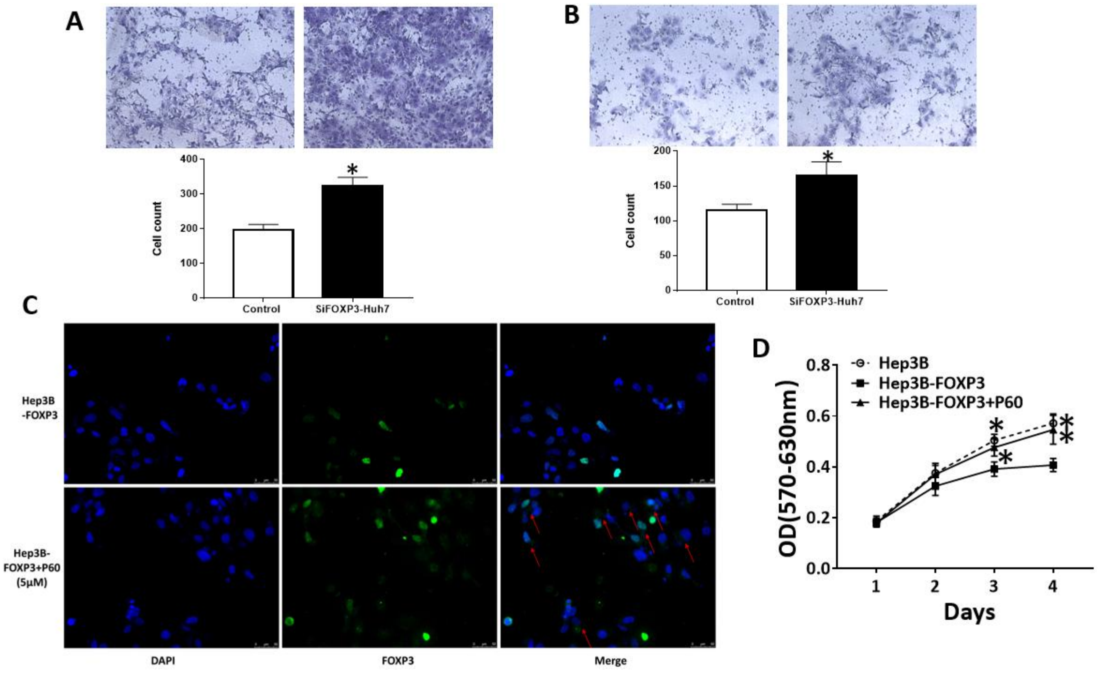

Figure S6. A The effect of FOXP3 knockdown on the migration of Huh7 cells. B The effect of FOXP3 knockdown on the invasion of Huh7 cells. FOXP3 knockdown promotes the migration and invasion abilities of Huh7 cells. C&D P60-mediated inhibition of FOXP3 nuclear translocation reduces the proliferation of HCC cells. C.

Immunofluorescence analysis of the subcellular location of FOXP3. The cells positive for FOXP3 were shown in green. The nucleus was stained with DAPI (blue). The merged fluorescence analysis showed that P60 could inhibit the translocation of FOXP3 into nucleus in FOXP3-postive Hep3B cells (red arrow). D. P60 reversed FOXP3-mediated inhibition of cell viability. FOXP3-positive Hep3B cells were treated with or without 5 μM of P60 for 1, 2, 3 and 4 days. At the end of the treatment, MTT was performed to determine the cell viability. *P<0.05.

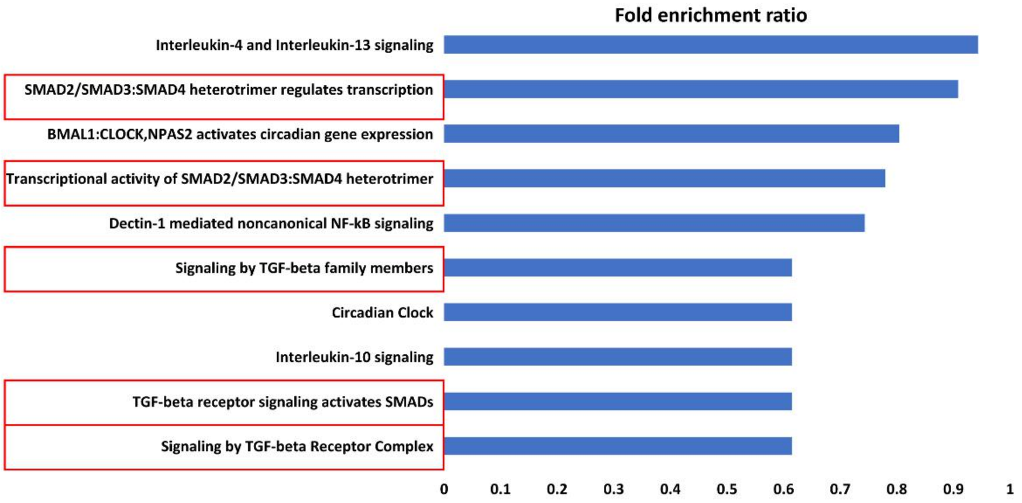

Figure S7. The pathways related to the differential expression gene. RNA-sequence was performed in Hep3B-FOXP3 cells and the control cells.
